# Stage 3 N2 Lung Cancer: A Multidisciplinary Therapeutic Conundrum

**DOI:** 10.1007/s11912-023-01486-2

**Published:** 2024-01-02

**Authors:** Lily Carter, Vedika Apte, Arushi Shukla, Aruni Ghose, Raj Mamidi, Alexandra Petohazi, Shania Makker, Soirindhri Banerjee, Stergios Boussios, Giuseppe L. Banna

**Affiliations:** 1 Division of Surgery, Cancer and Cardiovascular Medicine, Imperial College Healthcare NHS Trust, London, UK; 2 University College London Medical School, London, UK; 3 University College London Oncology Society, London, UK; 4 Barts and the London School of Medicine and Dentistry, Queen Mary University of London, London, UK; 5 School of Biosciences Education, Faculty of Life Sciences and Medicine, King’s College London, London, UK; 6 Barts and the London Oncology Society, London, UK; 7 Department of Medical Oncology, Barts Cancer Centre and Cardio-Oncology, Barts Heart Centre, St Bartholomew’s Hospital, Barts Health NHS Trust, London, UK; 8 Department of Medical Oncology, Medway NHS Foundation Trust, Gillingham, Kent, UK; 9 Department of Medical Oncology, Mount Vernon Cancer Centre, East and North Hertfordshire NHS Trust, London, UK; 10 Immuno-Oncology Clinical Network, Liverpool, UK; 11 Future Cancer Leaders, United Kingdom and Ireland Global Cancer Network, London, UK; 12 Health Systems and Treatment Optimisation Network, European Cancer Organisation, Brussels, Belgium; 13 Oncology Council, Royal Society of Medicine, London, UK; 14 Department of General Surgery, Lancashire Teaching Hospitals NHS Foundation Trust, Preston, UK; 15 University College London Cancer Institute, London, UK; 16 Department of Radiation Oncology, IPGMER and SSKM Hospital, Kolkata, India; 17 School of Cancer and Pharmaceutical Sciences, Faculty of Life Sciences and Medicine, King’s College London, London, UK; 18 Kent and Medway Medical School, University of Kent, Canterbury, UK; 19 AELIA Organisation, 9th Km Thessaloniki – Thermi, 57001 Thessaloniki, Greece; 20 Department of Medical Oncology, Portsmouth Hospitals University NHS Trust, Portsmouth, UK; 21 Faculty of Science and Health, School of Pharmacy and Biomedical Sciences, University of Portsmouth, Portsmouth, UK

**Keywords:** Non-small cell, Lung cancer, Neoadjuvant, Resectable, Immunotherapy, Radiotherapy, Chemotherapy

## Abstract

**Purpose of Review:**

The treatment of stage III N2 non-small cell lung cancer (NSCLC) remains debated. There is an absence of a universally agreed definition of resectability for this heterogeneous group and a lack of trial data.

**Recent Findings:**

We reviewed and compared current international guidelines and evidence surrounding management of stage III N2 NSCLC. The Irish and Australian guidelines advise subcategorising N2 disease into N2a (may be resectable) and N2b (never resectable). On the contrary, American and British guidelines avoid subcategorising N2 disease, emphasising importance of local MDT decisions. It is suggested that evidence for resection of stage III tumours is relatively weak, but that stage IIIA should generally be considered for resection, and stage IIIB is not recommended for resection. For resectable disease, surgery may be combined with neoadjuvant chemoimmunotherapy, or adjuvant chemotherapy followed by immunotherapy and radiotherapy in selected patients.

**Summary:**

There is some evidence that technically resectable disease can be treated solely with radiotherapy with similar outcomes to resection. In the event of unresectable disease, chemoradiotherapy has been the traditional management option. However, recent studies with chemoradiotherapy alongside immunotherapy appear promising. There are many factors that influence the treatment pathway offered to patients with stage III N2 NSCLC, including patient factors, team expertise, and local resources. Therefore, the role of MDTs in defining resectability and formulating an individualised treatment plan is crucial.

## Introduction

Determining the course of treatment for patients with stage III N2 non-small cell lung cancer (NSCLC), also known as locally advanced LC, remains a clinical challenge. Due to heterogeneity within this group, there are no standardised therapeutic protocols, rather the choice of treatment is dependent on multidisciplinary judgement and patient preference. Defining resectability forms the basis for treatment options. In resectable disease, surgery is the choice of treatment, with or without neoadjuvant chemotherapy, chemoradiotherapy, or chemoimmunotherapy. In some cases, adjuvant chemotherapy is also offered. Surgical excision aims to resect the tumour with R0 margins. For those with non-resectable diseases, the main choice of treatment is chemoradiotherapy with/without adjuvant immunotherapy [1•, 2]. It is hard to practise evidence-based medicine with this group, as existing studies demonstrate no significant increase in survival rate with one approach over the other. This reinforces the need for good clinical judgement to provide personalised care [3]. The interface between a surgical route and a medical therapy route is embedded in defining the realm of resectability. There is an absence of a universally agreed definition for the resectability of stage III NSCLC, meaning that geographical disparities exist [4]. This again emphasises the unmet need for standardisation of resectability and optimisation of treatment pathways.

LC is the most diagnosed cancer worldwide and is also the leading cause of cancer-related deaths [5]. Eighty-five of LCs worldwide are NSCLCs. Moreover, a significant proportion of NSCLC patients are diagnosed at stage III, which is associated with poor prognosis and survival rates. The 1-year survival rate is as low as 42.5%, with a 5-year survival rate between 10 and 40% [1•, 4]. Poor survival outcomes despite the availability of curative treatment should encourage advancements in standardising treatment protocols.

The differences in manifestations of this disease make it tedious to follow a single treatment pathway. However, other contributing factors impose a challenge in treating this heterogenous group. Between 20 and 35% of diagnoses are made at stage III of NSCLC [6]. The National Lung Cancer Audit (NLCA) report from 2023 states that 40% of patients diagnosed with stage III NSCLC were treated with palliative intent [7]. This reiterates the concern that too many cases are detected at a stage where it is too late for curative treatment. In addition, the mean age of diagnosis of locally advanced disease is between 65 and 79 years, a vulnerable population where biopsychosocial factors (e.g. comorbidities, frailty, social isolation, health stigma) may impact psychological distress and quality of life outcomes, arguably more so than a younger population [8]. It is therefore important to recognise the complexity of factors that may impact prognosis, as such knowledge can be used to make informed decisions for personalised and suitable treatments.

## Aims and Objectives

Through this narrative review, we aim to compare the treatment options offered in stage III N2 NSCLC to develop a comprehensive understanding of how to manage patients of this heterogeneous group. We aim to assess to what extent can surgical management be effective, and how neoadjuvant or adjuvant therapies can help in treating the disease.

The objectives below describe the steps taken to ensure our review is effective in informing clinicians of the range of treatment pathways adopted currently, and a detailed analysis of those options to allow for better evidence-based decisions in practise. To achieve our aim, our objectives were as follows:

1. Define the stage III N2 NSCLCs, breaking down the subtypes to understand why this disease is known to be heterogeneous.
2. Examine international guidelines on management of NSCLC and compare how they define resectability of stage III N2 NSCLC, and how this impacts management.
3. Identify trials conducted for this patient group and analyse the patient outcomes to assess which methods of treatments are seen to be effective—including surgical resection, chemotherapy, radiotherapy, and immunotherapy.

## Methodology

To outline what treatments are offered, the international guidelines of the following nations were summarised: England, The USA, Ireland, and Australia. All data used was done so using trusted sources used by doctors of the respective nations. EMBASE and MEDLINE searches were conducted between February and April 2023, and clinical trials investigating treatments of stage III N2 NSCLC were included. This topic is heavily debated in the literature. The primary search phrase ‘stage III N2 NSCLC’ was used, including keywords such as ‘treatment’, ‘randomised controlled trials’, ‘surgical resection’, ‘neoadjuvant therapy’, ‘radiotherapy’ and ‘immunotherapy’.

## Defining Stage III N2 NSCLC

Stage III tumours, particularly those with N2 spread, are a heterogeneous group with unclear treatment pathways and a lack of a universally agreed definition of resectability [4]. This review is focused on stage IIIA and stage IIIB tumours with N2 spread. This is defined by tumour size ranging from < 3 to > 7 cm, which may invade a main bronchus, the visceral pleura, the parietal pleura, the chest wall, the phrenic nerve or the parietal pericardium, and may have > 2 tumour nodules in one lung lobe. N2 spread is defined by spread to ipsilateral mediastinal or subcarinal lymph nodes. It has been suggested that further definition of lymph node status is helpful for prognostication and treatment planning. N2 can be further split into N2a1 (single zone, non-bulky lymph node spread, no skip metastases), N2a2 (single zone, non-bulky lymph node spread, with skip metastases), or N2b (multiple zone and/or bulky lymph node spread) [9]. Stage IIIC disease is characterised by N3 spread and therefore is not considered here.

Stage III N2 NSCLC tumours encompass several stages of tumour under the TNM classification system (Table 1) [10].

**Table 1:** TNM stages of stage III N2 NSCLC tumours [10]

AJCC stage	Stage grouping	Description
IIIA	T1a/T1b/T1c N2 M0	T1: The cancer is < 3 cm, has not invaded the pleura, and does not affect the main bronchus N2: The cancer has spread to ipsilateral subcarinal or mediastinal lymph nodes M0: There are no distant metastases
	T2a/T2b N2 M0	T2: The tumour is 3–5 cm in size OR the tumour is < 5 cm and has invaded the main bronchus, invaded the visceral pleura, or there is atelectasis/postobstructive pneumonitis extending to the hilum N2: The cancer has spread to ipsilateral subcarinal or mediastinal lymph nodes M0: There are no distant metastases
IIIB	T3 N2 M0	T2: The tumour is 5–7 cm in size OR the tumour has invaded the chest wall, the parietal pleura, the phrenic nerve, or the parietal pericardium N2: The cancer has spread to ipsilateral subcarinal or mediastinal lymph nodes M0: There are no distant metastases

## Resectability vs Operability

It is worth discussing the distinction between operability and resectability here. Resectability looks at the tumour itself to assess whether surgical resection is technically possible. Operability takes account of other factors to assess the suitability of individual patients for surgical intervention. Assessing resectability and operability necessitates assessment of tumour-specific factors (cell type, distant metastases, lymph node invasion), patient-specific factors (age, comorbidities), and surgery-specific factors (type of surgical procedure, whether it is elective or emergency, local resources including skill and experience of surgeons and anaesthetists). Patients are categorised as either medically operable or medically inoperable, and technically resectable or technically non-resectable—for an operation to be considered, patients must be medically operable and the tumour technically resectable [11].

One factor affecting the operability of patients with lung tumours is the tendency towards comorbidity. This is, in part, due to the high proportion of lung cancer patients being smokers or ex-smokers. As well as increased risk of malignancy, these patients are also more likely to have other respiratory diseases (e.g. chronic obstructive pulmonary disease, interstitial lung disease), as well as cardiovascular conditions (e.g. peripheral vascular disease, ischaemic heart disease, ischaemic stroke, hypertension) as a result of their smoking history. Increasing age is also a risk factor for lung cancer, and elderly patients are more likely to have other coexisting chronic conditions (e.g. chronic kidney disease, diabetes mellitus) and be altogether more frail than younger patients [12]. One study found that of patients with a first diagnosis of lung cancer, 52.5% had COPD, 15.7% had diabetes, and 12.9% had congestive heart failure. The study found that the presence of comorbidities negatively affects the survival of lung cancer patients [13]. A variety of tools can be used as part of an assessment to decide on operability based on patient factors. This includes Thoracoscore, AAC/AHA risk stratification, lung function testing, and cardiology review [14].

## International Guidelines for Defining Resectability of Stage III N2 NSCLC

The guidelines considered here are limited to those published in English that have been published or updated within the past 5 years.

### National Cancer Institute (USA)

The Non-Small Cell Lung Cancer Treatment (PDQ®)–Health Professional Version from the National Cancer Institute states that stage IIIA may be considered for surgery, and that stage IIIB is never a candidate for surgery [15]. Tumours are categorised into three broad categories—(1) surgically resectable disease (includes selected stage III tumours), (2) locally (T3–T4) and/or regionally (N2–N3) advanced disease, and (3) distant metastatic disease. It states that select patients with N2 disease (and T3 disease) can be considered for resection alongside neoadjuvant/adjuvant chemo/radiotherapy. Other specific stage IIIA tumours defined as being candidates for surgery are superior sulcus tumours (Pancoast tumours) and tumours that invade the chest wall. The importance of lymph node evaluation for accurate staging and resulting increased survival is highlighted, but this relates only to stage I disease, and no evidence pertaining to stage III N2 disease is included. CT, MRI, and PET imaging, as well as invasive staging techniques, are discussed, and the importance of identifying metastatic disease accurately to avoid unnecessary surgery. This suggests that accurate staging is an important step in deciding on resectability of the tumour; however, once a tumour is deemed to be metastatic, it would no longer be classified as stage III, so this does not help decide on resectability of stage III N2 disease. Treatment options for resectable tumours are highlighted, in terms of neoadjuvant/adjuvant chemo/radiotherapy, but there is no mention of how to initially categorise whether those stage IIIA N2 tumours are resectable or not. The evidence referenced is not recent (2005, 2009, 2011) and largely focuses on the benefits of surgery and surgical technique regarding lymph node sampling vs resection. The evidence frequently highlights that current treatment options are not satisfactory, and the importance of recruiting patients for clinical trials. The evidence used for this guideline is frequently from old trials and data, and a higher volume of new evidence is clearly needed to guide further improvement of guidelines. Immunotherapy is mentioned as an option for the treatment of resectable and unresectable tumours, but the evidence suggests there is no increase in overall survival.

### NICE (England)

The National Institute for Clinical Excellence (NICE) produced a guideline in 2019 which does not make any definitive recommendations on how to assess resectability of stage IIIA N2 NSCLC. However, it does state that “for people with operable stage IIIA–N2 NSCLC who can have surgery and are well enough for multimodality therapy, consider chemoradiotherapy with surgery” [16]. There is no mention of surgery as an option for stage IIIB disease, discussing radical radiotherapy only. There are some similarities with the US guideline, including emphasising ruling out metastasis before embarking on curative treatment pathways. There are also frequent mentions of the importance of multidisciplinary care—“Multidisciplinary teams that provide chemoradiotherapy with surgery should have expertise in the combined therapy and in all of the individual components” [16]. NICE suggests that decisions about resectability and operability should be left up to the MDT looking after patients, rather than relying on national guidelines. However, it does state that, from the evidence, chemoradiotherapy and surgery are the most effective treatments and should be used preferentially to both chemoradiotherapy alone and chemotherapy and surgery, where suitable. It states that the current trend is not to treat in this way, so this guideline may alter and improve current practise. Regarding immunotherapy, the guidance recognises the likely benefits, but states that the evidence for the cost and clinical effectiveness of immunotherapy alongside surgery is lacking, and recommends further research is done.

### Northern Cancer Alliance (Northeast England and North Cumbria)

A second English guideline, reviewed and updated in 2023, from the Northern Cancer Alliance is more specific about what should and should not be considered. This may be because the regional teams have agreed on what is achievable for their region, based on the experience of staff and resources. It states that those with stage IIIA N2 disease should not be offered surgery initially but may be reconsidered for surgical resection following chemotherapy [17]. There is no discussion of the role that immunotherapy may play.

### Irish Department of Health

The 2017 Irish guidelines for LC management were published in 2017 but reviewed and updated in 2023 [18]. The guidelines state that surgery should be considered as part of treatment for T1–3 N2 disease, provided lymph node involvement is non-fixed, non-bulky, and single zone. Reference is made here to further separate lymph node disease into N2a and N2b, as mentioned above [9], recognising that the extent of lymph node disease has more of an impact on the outcome than which lymph node(s) is/are affected. Patients should also be able to tolerate multimodality therapy, as surgery must be accompanied by chemotherapy and/or radiotherapy. There is no discussion of immunotherapy. Those with N2b disease should not be considered for surgery. This recommendation is based on evidence also used in the BTS guidelines [14] and the Scottish Intercollegiate Guidelines Network (SIGN) guidance, published in 2014. The guideline discusses the benefits seen in surgical intervention for stage IIIA N2 patients in the literature but recognises the relative weakness of these studies, which have small sample sizes and are retrospective case series [18, 19]. The SIGN guidance was due for review in 2017 but no updates appear to have been published since, and as such will not be considered independently here. Stage IIIB is not discussed in the section looking at surgical intervention but is discussed alongside stage VI in sections on treatment for advanced cancers, which does not include surgery, suggesting stage IIIB is not treated with surgery.

### Cancer Council Australia

The guidelines from the Cancer Council Australia, last modified in 2018, have a specific section addressing the issue of defining the operability of stage III disease. Operability is discussed concerning patient factors and tumour factors. Tumours that may be unresectable include those with contralateral lymph nodes, N3 involvement, and most T4 tumours [20]. Contralateral lymph nodes would also be categorised as N3; however, the only T4 tumour classified as stage III is N2 T4N2M0, which is stage IIIB [10]. This suggests that stage IIIB N2 tumours are unlikely to be resectable. Other considerations when assessing resectability include “nodal size, number of stations involved, extracapsular extension and involvement of the recurrent laryngeal nerve” [20]. Those with N2 disease are recommended to have multimodality treatment—chemotherapy, surgery, and radiotherapy—not immunotherapy. Evidence for the final recommendations is from a 2009 study. The evidence-based recommendation is: “Unselected patients with biopsy confirmed stage IIIA (N2) disease are best treated with chemoradiotherapy alone” [20]. However, the practise point (recommendations made by an expert opinion that are outside the scope of the systematic review) states that “Induction chemoradiotherapy followed by surgery in selected patients with cIIIA (N2) disease is feasible and improves progression-free survival. Provided the patient does not require a pneumonectomy, the addition of surgery may improve overall survival.” These recommendations were last reviewed in 2015, despite the guidelines themselves being updated in 2018.

### Conclusion of Guidelines Regarding Categorising Stage III N2 Tumours as Resectable or Non-resectable

Whilst there are some differences between these guidelines, there are some similarities. They are largely all based on similar data, and there are a limited number of trials in this area. N2 disease does not exclude patients from surgical intervention, and the Irish and Australian guidelines mention categorising N2 disease into (1) N2a or single zone/non-bulky/non-fixed and (2) N2b or bulky/fixed/multizone. They state that N2a is possibly resectable whereas N2b is not. The Australian guidelines also include involvement with the recurrent laryngeal nerve as something to be considered when assessing resectability. The US and NICE guidelines, however, avoid categorising N2 disease resectability, stating the importance of the local/regional MDT and specialist teams in making these decisions. This is reflected in the guidelines from the Northern Cancer Alliance, which states that IIIA disease should not initially be treated with surgery, but considered once chemotherapy has been given. This contrasts with the NICE guideline which concluded from the evidence that there is no benefit to treating patients with chemotherapy and surgery over chemoradiotherapy, but that the most effective treatment is chemoradiotherapy and surgery. All the guidelines allude to the fact that evidence for resection of stage III tumours is relatively weak, but that stage IIIA can be considered for resection. Stage IIIB is not recommended for resection in any of the guidelines (Table 2). There is little mention of immunotherapy as a treatment option and no strong recommendations for its use [15–18, 20].

**Table 2:** Summary table of international guidelines pertaining to resectability of stage III N2 NSCLC

Guideline	Stage IIIA N2a	Stage IIIA N2b	Stage IIIB N2
National Cancer Institute (USA)	Possibly resectable		Not resectable
NICE	Possibly resectable		Unlikely resectable
Northern Cancer Alliance	Possibly resectable after chemotherapy		Unlikely resectable
Irish Department of Health	Possibly resectable	Not resectable	Unlikely resectable
Cancer Council Australia	Possibly resectable after chemoradiotherapy		Unlikely resectable

## Treatment Strategies

### The Role of the Multidisciplinary Team (MDT)

In the management of stage III N2 NSCLC, the multidisciplinary team (MDT) plays a pivotal role in decision-making. This is reflected in the emphasis that several of the guidelines discussed above place on the MDT. Mainguene et al. [21] examined the reproducibility of MDT decisions concerning medical strategy versus surgical resection for stage IIIA/B-N2 NSCLC patients. Over a 2-year period, 30 cases meeting the criteria were discussed in MDT meetings. Results indicated that 44% of patients were recommended medical strategies, whilst 56% were advised surgical resection. Importantly, re-discussion of cases yielded consistent treatment decisions in 70% of instances, and conflicting decisions did not impact overall survival [21].

## Resectable Disease

### Neoadjuvant Chemoimmunotherapy Followed by Surgery

Neoadjuvant therapy in the context of resectable NSCLC may involve chemotherapy or immunotherapy. However, reports from more recent randomised control trials suggest that it may be largely more beneficial to use both, rather than one alone, in a neoadjuvant setting. Six distinct randomised control trials look for the added benefit, efficacy, and safety of neoadjuvant chemoimmunotherapy followed by adjuvant immunotherapy compared with neoadjuvant chemotherapy alone. The results of these trials are shown in Table 3. Pathological complete response (pCR) was a common endpoint for all the trials and has therefore been included for comparison.

**Table 3:** Summary table for resectable disease

Reference	Intervention	Inclusion criteria	Number of subjects	Progression-free survival (median or %)	Overall survival (median)	Pathological complete response (%)	Event-free survival/relapse-free survival (median or %)	X-year survival (%)
Neoadjuvant chemoimmunotherapy followed by surgery								
(Forde et al., [22])	Nivolumab + platinum-based chemotherapy	Resectable stage IB to IIIA NSCLC	179	-	-	24.0%	EFS—31.6 months	-
	Platinum-based chemotherapy		179	-	-	2.2%	EFS—20.8 months	-
(Wakelee et al., [23•])	Pembrolizumab + chemotherapy	Resectable stage II, IIIA, or IIIB (N2 stage) NSCLC	397	-	80.9% (2 years est.)	18.1%	EFS—62.4% (2 year)	-
	Placebo + chemotherapy		400	-	77.6% (2 years est.)	4.0%	EFS—40.6% (2 year)	-
(Provencio et al., [24•])	Nivolumab + platinum-based chemotherapy	Resectable stage IIIA or IIIB NSCLC	57	67.2% (2 years est)	85.0% (2 years est)	37.0%	-	-
	Platinum-based chemotherapy		29	40.9% (2 years est)	63.6% (2 years est)	7.0%	-	-
(Heymach et al., [25])	Durvalumab + platinum-based chemotherapy	Resectable stage II–III NSCLC	401	-	-	17.2%	-	-
	Placebo + platinum-based chemotherapy		401	-	-	4.3%	EFS—25.9 months	-
(Lei et al., [26])	Camrelizumab + chemotherapy	Resectable stage IIIA or IIIB (T3N2) NSCLC	43	-	-	32.6%	-	-
	Chemotherapy		45	-	-	8.9%	-	-
(Lu et al., [27])	Toripalimab + chemotherapy	Resectable stage II–III NSCLC	202	-	-	24.8%	-	-
	Placebo + chemotherapy		202	-	30.4 months	1.0%	EFS—15.1 months	-
Neoadjuvant chemoradiotherapy and surgery ± adjuvant chemotherapy								
(Furrer et al., 2021)	Non-extended resection	T3/4 locally advanced						
NSCLC including single or multilevel N2	123	-	54.2% (3 years)	13.2%	-	-		
	Extended resection		36	-	61% (3 years)	-	-	-
(Tanaka et al., [28])	Neoadjuvant chemotherapy with concurrent radiotherapy (50 Gy), followed by surgical resection and adjuvant chemotherapy	Resectable stage IIIA-N2	40	63% (2 years)	75% (2 years)	29% (10 of 34 operated on)	RFS—62% (2 years)	-
Neoadjuvant chemotherapy, radiotherapy vs resection								
(Johnstone et al., [29])	Preoperative chemotherapy and surgical resection	Patients with stage IIIA (T1-T3N2M0) NSCLC	29	14 months	19.4 months	-	-	22% (4 years)
	Chemotherapy + radiotherapy		32		17.4 months	-	-	22% (4 years)
(van Meerbeeck et al., [30])	Induction chemotherapy + resection	Stage IIIA-N2 NSCLC	167	9 months	16.4 months	-	-	15.7% (5 years)
	Induction chemotherapy + radiotherapy		165	11.3 months	17.5 months	-	-	14% (5 years)
(Albain et al., [31])	Radiotherapy + chemotherapy + surgical resection	Stage T1-3pN2M0 NSCLC	202	12.8 months	23.6 months	-	-	27.2% (5 years)
	Radiotherapy + chemotherapy		194	10.5 months	22.2 months	-	-	20.3% (5 years)
BMLD vs SLND								
(Kużdżał et al., [32])	Bilateral mediastinal lymphadenectomy	Stage I–IIIA NSCLC patients	89	90.5 months	72% (5 years)	-	RFS—85 months	-
	Systematic lymph node dissection			157 months (est.)	53% (5 years)	-	RFS—156 months (est.)	-

As the results are statistically significant, we can safely infer that neoadjuvant chemoimmunotherapy greatly improves the pathologic response rate compared to chemotherapy alone. This is also true for the other primary/secondary endpoints, such as event-free survival, major pathological response, and overall survival meaning that this form of dual neoadjuvant therapy can increase treatment outcomes in the long run. This greatly challenges previous therapeutic choices for this disease setting. Furthermore, across the trials, treatment-related side effects, including Grade 3 to 5 adverse events, were almost similar in both treatment arms, meaning that the addition of immunotherapy to the neoadjuvant regime still produces quite manageable toxic effects whilst increasing pCR [22, 23•, 24•, 25–27].

### Neoadjuvant Chemoradiotherapy and Surgery ± Adjuvant Chemotherapy

A prospective study by Furrer et al. was carried out across three medical centres, focusing on the sequential approach of induction chemoradiotherapy followed by surgical resection. It compared anatomical resection with extended resection. The chemoradiotherapy regimen incorporated cisplatin, docetaxel, and radiation (44 Gy in 22 fractions over 3 weeks). Among the 197 patients enrolled, 38 were excluded due to progressive disease. The remaining 159 patients had anatomical resections with systematic lymphadenectomy. Of these 159 patients, 36 had extended resections. Eighty percent of patients achieved an R0 status after anatomical lung resections; this was similar to whether the resection was extended or not. The 3-, 5-, and 10-year survival following extended and non-extended resection were similar—61%, 45%, 29.5% and 54.2%, 45.7% 26.8%, respectively. Achievement of R0 status following resection had the most impact on progression-free survival and overall survival [33].

Tanaka et al. conducted a multiinstitutional study in 2018, aiming to examine the impact of induction chemotherapy and concurrent thoracic radiotherapy (50 Gy) followed by resection and postoperative consolidation chemotherapy on IIIA-N2 NSCLC patients. Forty patients received initial chemotherapy with carboplatin and paclitaxel and radiotherapy, from which there was a 58% overall response rate. Thirty-four of these patients went on to have surgical resection, and 20 patients were able to complete all 3 treatments. Thirty-two of the 34 patients operated on achieved complete resection (R0), meaning a total of 80% of patients from the study achieved R0. Progression-free survival at 2 years was 63%. The authors highlighted bronchopleural fistula (BPF) as an adverse outcome reported in 3 patients, possibly due to the higher dose of thoracic radiotherapy used in this study before surgery. The use of bronchial stump coverage is suggested to try to reduce the risk of this complication [28].

A meta-analysis conducted by Zhao et al. evaluates the ideal treatment for stage III N2 NSCLC by comparing the three main treatment modalities (surgery, chemotherapy, and radiotherapy). It concludes that neoadjuvant chemotherapy, then surgery, followed by adjuvant chemotherapy/radiotherapy has the most potential to be the best treatment option in terms of the highest overall survival and lowest treatment-related deaths [34]. This is not included in the table below as it is not a trial.

### Neoadjuvant Chemotherapy, Followed by Radiotherapy vs Resection

In stage III NSCLC, where there is a controversy between the use of surgery vs other treatment modalities, it may potentially be more or just as suitable to use radiotherapy without surgical resection in resectable disease. The following 3 studies are in relation to this: (1) phase III trial conducted by RTOG and ECOG (USA), looking at radiotherapy and chemotherapy vs neoadjuvant chemotherapy and surgery [29]; (2) randomised control trial conducted by the Dept. of Respiratory Medicine in University Hospital Ghent (Belgium), analysing surgery vs radiotherapy postinduction chemotherapy [30]; (3) phase III trial conducted in the USA and Canada, comparing radiotherapy after chemoradiation with or without surgical resection [31]. In all three trials, the treatment outcomes were very similar between each paper’s treatment arms, implying that surgery provides no added benefit to survival and that radiotherapy without surgery could be considered a viable treatment option for stage III N2 NSCLC. The results are accumulated in Table 3. Generally, any differences between treatments were either very small or not statistically significant, meaning that both surgery and radiotherapy are suitable treatment options—surgery is not necessarily more effective.

However, whilst these trials suggest that radiotherapy is a fitting option, they do not imply the superiority of radiotherapy, i.e. it is not better than using surgery, because there is virtually no difference in survival between the two across the trials. There may be fewer treatment-related deaths with radiotherapy compared to surgery [29, 31] (4 vs 16 patients and 1 vs 2 patients, respectively), with the main cause of radiotherapy-related deaths being radiation pneumonitis. Nonetheless, there is also a risk of radiotherapy noncompliance due to the associated toxicities, which can affect any organ in the irradiated region including the skin, heart, and oesophagus as well as healthy lung tissue. These effects are also graded from 1 to 4 [35], with grade 3/4 toxicities seen in patients in the study by van Meerbeeck et al. [30]. Because of these radiation-related side effects, for patients with stage III N2 NSCLC that are suitable for both surgical resection and radiotherapy, the appropriate treatment option could be chosen based on factors that may indicate the patient’s ability to tolerate these side effects. For example, if the patient is older and frailer, it may be wiser to opt for surgery as they may not tolerate the side effects, risking treatment noncompliance. However, this sparks a different debate, as older patients are less likely to tolerate a major surgery like tumour removal, and perhaps more research needs to be conducted to create a guideline regarding which treatment modality to go for if both are deemed equally appropriate for the patient in terms of treatment outcomes.

The debate between surgery and radiotherapy also extends to early-stage, resectable NSCLC, and perhaps the results of such studies could be extrapolated and applied to resectable stage III disease as well. Even for early-stage NSCLC, studies claim the non-inferiority of using radiotherapy as a single treatment modality when compared with surgery [36••]. An article in The Lancet Oncology by Louie et al. illustrates exactly this by looking at the revised results of the STARS trial. Originally, the STARS and ROSEL studies were halted prematurely and statistically significant conclusions could not be drawn due to a small sample size [37]. The revised STARS trial’s cohort consists of 80 patients, with virtually no differences in overall survival, progression-free survival, local recurrence, and distant metastases between the two treatment arms. However, a higher regional recurrence rate was noted in the radiotherapy group, but the trial also shows that any patients who might have suffered from recurrence were effectively treated with chemoradiation. Such patterns were not reported postsurgery and would be helpful to know for a true comparison [38]. A BMJ article by Khakwani et al. identifies some issues with referring patients for radiotherapy alone—it notes considerable delays in the receipt of radiotherapy, which could negatively impact treatment outcomes [39]. A potential reason for this delay may be the fact that patients have to be referred to specific commissioned centres to receive treatment, as not all radiotherapy centres in the UK can administer the stereotactic radiation required for NSCLC [38, 39]. Maybe this indicates that rather than radiotherapy being ineffective as a treatment form, the provision of the service itself needs to be more streamlined. Whilst surgical methods are rapidly advancing, with the growth of minimally invasive methods, the debate between surgery and radiotherapy remains—but if early-stage NSCLC can effectively be treated with radiotherapy, why not stage III? More research and clinical trials would need to be conducted to answer this, but perhaps for now treatment should be given based on the patient’s treatment capability as well as careful MDT discussions.

### Other Surgical Considerations

#### Bilateral Mediastinal Lymph Node Dissection vs Systematic Lymph Node Dissection

A randomised control trial to evaluate the influence of bilateral mediastinal lymph node dissection (BML) versus systematic lymph node dissection (SLND) on survival among stage I–IIIA NSCLC patients undergoing surgical resection was conducted by Kużdżał et al. Among 102 selected patients, complications did not significantly differ between the two groups, though BML necessitated a longer operative time (24 vs. 14). The 5-year survival rate was on average 72% for the BML group and 53% for the SLND group. This difference was most stark in the LLL group (90.4% vs 37.5%), followed by the RLL group (90% vs 50%) and then the LUL group (75% vs 58.8%). The inverse was true for those patients with RUL/RML tumours (36.4% vs 57.1%) [32].

#### Robot-Assisted Thoracoscopic Surgery vs Thoracotomy

A randomised control trial explored the short-term outcomes of robot-assisted thoracoscopic surgery (RATS) compared to thoracotomy in treating cN2 stage NSCLC patients. Among the 113 patients diagnosed, 108 were selected and divided into RATS (*n* = 58) and thoracotomy (*n* = 55) groups. Radical lobectomy with mediastinal lymph node dissection was conducted in all cases. RATS demonstrated superiority in terms of reduced postoperative complications, thorough lymph node dissection, diminished blood loss, and shorter operative time. The issue of unplanned conversion to thoracotomy, although rare, remains a consideration. As this study did not evaluate the long-term impacts of RATS compared with thoracotomy, it is difficult to compare with other interventions (and hence is not included in the summary table below) [40].

## Unresectable Disease

### Chemoradiotherapy Alone

A guideline by Okawara et al. is based on the evidence seen in two meta-analyses of randomised control trials. It identifies the survival benefit of using cisplatin-based chemotherapy as well as radiotherapy, rather than radiotherapy alone, with a 13% reduction in the risk of death at 2 years using combined treatment in both meta-analyses [41]. A trial by Giorgio et al. looks at the elderly population in particular as the optimal management for them has not been specified to date. It suggests that the safest management for them involves concurrent chemoradiotherapy after induction chemotherapy [42]. The multicentre phase II trial investigates the feasibility of induction chemotherapy followed by concurrent chemoradiotherapy (weekly cycles) in terms of toxicity and overall survival. It concludes that this is an appropriate method of administration, one with moderate toxicities, with 36 out of 64 patients suffering from grade 1/2 oesophagitis and much smaller numbers with grade 3/4 oesophagitis and grade 1/2 pneumonitis. An overall response rate of 74.6%, a median overall survival of 461 days (15.2 months), and a median time to progression of 247 days (8.1 months) were also noted [43].

However, the multicentre trial also recognises the challenge of toxicity as a concurrent approach would lead to more toxicities than a sequential approach. As well as the ones mentioned previously, other side effects include febrile neutropenia, with a potential complication of neutropenic sepsis [44]. This suggests a sequential approach for patients who have poorer performance statuses or poorer lung function, for example, may be preferable. All in all, these papers suggest that concurrent chemoradiotherapy is better than both radiotherapy alone and sequential chemoradiotherapy, as concurrent use reduces the risk of death, even in elderly patients. As of now, for patients susceptible to more severe toxicities, perhaps radiotherapy alone or sequential chemoradiotherapy is the preferred option, but in the future, following more scientific advancements, if less toxic radiation techniques and chemotherapy drugs came about, then concurrent therapy could be used for all stage III N2 NSCLC patients, regardless of susceptibility.

### Chemoradiotherapy and Immunotherapy

After radical chemoradiotherapy for resectable stage III N2 NSCLC, patients may be given adjuvant immunotherapy, according to international guidelines [4]. Precision cancer medicine is rapidly evolving, and the use of immunotherapy in the management of this disease, whether that is with chemoradiotherapy or surgery, is being implemented more and more internationally. So what is immunotherapy? It is essentially the use of the body’s innate and adaptive immune systems to strike a balance between destroying cancer cells and not causing autoimmune responses [45]. Approved immunotherapeutic agents for NSCLC include immune checkpoint inhibitors such as nivolumab, pembrolizumab, durvalumab, and atezolizumab [46].

The phase III PACIFIC trial evaluates the use of immunotherapy in combination with chemoradiotherapy in unresectable disease. This trial looks at how beneficial the use of durvalumab (an anti-PDL-L1 antibody) is after radical chemoradiotherapy in patients with unresectable stage III N2 NSCLC when compared to a placebo group. In all assessed treatment outcomes, the durvalumab group (*n* = 197) proved to be superior to the placebo (*n* = 90). The median overall survival was 47.5 months in the durvalumab group vs 29.1 months. Median progression-free survival was 17.2 vs 5.6 months. The 4-year overall survival was 49.6% vs 36.3% and 4-year progression-free survival was 35.5% vs 19.5%. In terms of toxicities, 36.9% of patients in the durvalumab group experienced any-grade pneumonitis/radiation pneumonitis, compared to 27.0% in the placebo [47, 48]. Durvalumab is an anti-PDL-L1 antibody hence the assumption is that this treatment is only suitable for tumour cells that express PD-L1. However, a different article by Antonia et al. looking at the PACIFIC trial as well suggests that irrespective of the baseline PD-L1 expression, durvalumab may be an effective therapeutic agent [49]. All in all, the PACIFIC trial results illustrate the supposed added benefit of using immunotherapy, particularly durvalumab, in unresectable disease, and given the benefit, the conduction of more clinical trials specifically on resectable stage III N2 disease is warranted.

A literature review by Bassanelli et al. analyses radiotherapy and immunotherapy as an effective combination therapy for both early-stage and advanced NSCLC [50]. The article looks at different trials including the phase II PEMBRO-RT [51] and phase II LUN 14–179 [52] trials to name a few. The PEMBRO-RT trial shows that median progression-free survival was 1.9 months in pembrolizumab alone vs 6.6 months with pembrolizumab after radiotherapy, and median overall survival was 7.6 months vs. 15.9 months, respectively [51]. The LUN 14–179 trial looks at patients with unresectable stage III NSCLC and the use of pembrolizumab after chemoradiotherapy. The median time to metastatic disease or death was 30.7 months, the median progression-free survival was 18.7 months, and the median overall survival was 35.8 months. The 1-, 2-, and 3-year overall survival estimates were 81.2%, 62.0%, and 48.5% respectively. Grade 2 or more pneumonitis was recorded in 17.2% of patients [52]. The major takeaway from this article is that an ideal therapeutic strategy uses sequential radioimmunotherapy rather than immunotherapy alone and that the benefits of immunotherapy can be seen in both early-stage and advanced NSCLC. Sequential therapy is better not only because it improves treatment outcomes, but also because the interaction between radiation and the immune system can prevent immunotherapy resistance by overcoming the mechanisms by which this occurs. The article identifies the issue of immunotherapy resistance via checkpoint escape mechanisms, and the use of radiotherapy prior can help tackle this problem, which in turn improves outcomes.

As with chemoradiotherapy, the question that now emerges is how should immunotherapy be administered—concurrently or sequentially? The phase II DETERRED trial analyses the safety of concurrent triple therapy (chemoradioimmunotherapy) vs sequential therapy. It concludes that the concurrent atezolizumab with chemoradiotherapy is safe without any added toxicities when compared to sequential therapy as grade 3 + adverse events were seen in 57% of patients vs 60% respectively. In both groups, pneumonia was the most common grade 3 + adverse event [53]. An updated efficacy analysis of this trial shows that median progression-free survival for concurrent vs sequential atezolizumab was 15.1 vs 18.9 months, highlighting a comparable efficacy between the two regimes [54••]. The completed phase II NICOLAS study is another trial looking at the efficacy as well as safety of nivolumab with chemoradiotherapy in stage III NSCLC. The results are as follows: the 1-year and median progression-free survivals were 53.7% and 12.7 months respectively; after an extended follow-up, median overall survival was 38.8 months and 2-year overall survival was 63.7% [55••]. Based on the results of these two trials, the conclusion that concurrent triple therapy is safe and effective can be made (as progression-free survival in the NICOLAS trial is > 45%), and that this treatment strategy could be implemented more in clinical practise.

To summarise, immunotherapy is a potent treatment modality in NSCLC, as shown by the results of various trials (Table 4), and it should ideally be implemented more in patients deemed suitable. However, to build on this further, it would be useful to know whether chemotherapy in particular has any added benefit to radioimmunotherapy. Perhaps it should be excluded entirely because of its toxicities, but this would depend on how advantageous this treatment is in the first place. A potential trial could look at triple therapy vs radioimmunotherapy in stage III (N2) NSCLC patients.

**Table 4:** Summary table for unresectable disease

Reference	Intervention	Inclusion criteria	Number of subjects	Progression-free survival (median or %)	Overall survival (median)	X-year survival (%)
Chemoradiotherapy						
(Giorgio et al., [42])	Paclitaxel + carboplatin + radiotherapy	Age > 70 with unresectable stage IIIA N2 (bulky) or IIIB NSCLC	30	8.7 months	15 months	20% (3 years)
(Tell et al., [48])	Chemotherapy followed by concurrent chemoradiotherapy	Unresectable Stage III NSCLC	64	-	461 days	-
Chemoradiotherapy and immunotherapy						
(Senan et al., [47])	Chemoradiotherapy + durvalumab	Unresectable, stage III NSCLC + no disease progression after 2 or more cycles of platinum-based chemoradiotherapy	197	17.2 months	47.5 months	49.6% (4 years)
	Chemoradiotherapy + placebo		90	5.6 months	29.1 months	36.3% (4 years)
(Antonia et al., [49])	Chemoradiotherapy + durvalumab	Unresectable, stage III NSCLC + no disease progression after 2 or more cycles of platinum-based chemoradiotherapy	473	16.8 months	47.5 months	55.9% (1 year) 44.2% (1.5 years)
	Chemoradiotherapy + placebo		236	5.6 months	29.1 months	35.3% (1 year) 27.0% (1.5 years)
(Theelen et al., [51])	Pembrolizumab alone	Advanced NSCLC	40	1.9 months	7.6 months	-
	Pembrolizumab + radiotherapy		36	6.6 months	15.9 months	-
(Durm et al., [52])	Chemoradiation + pembrolizumab	Unresectable stage III NSCLC	93	18.7 months	35.8 months	81.2% (1 year) 62.0% (2 years) 48.5% (3 years)
(Liu et al., [54••])	Chemoradiation + consolidation atezolizumab	Unresectable locally advanced NSCLC	10	18.9 months	26.5 months	47.4% (4 years)
	Concurrent + consolidation atezolizumab		30	15.1 months	Not reached	63.7% (2 years)
(Peters et al., 2020)	Platinum-based chemotherapy + radiotherapy + nivolumab	Unresectable stage IIIA–IIIB	73	53.7% (1 year) 12.7 months (median)	38.8 months (median)	

## Conclusion

The toxicity of radiotherapy, immunotherapy, and chemotherapy, and the risks associated with surgery, in what is often a very comorbid and frail population with late-stage disease, make patient-centred care essential. Some attempts have been made in the guidelines to define what is categorised as resectable and non-resectable; however, there is an emphasis on the importance of local MDTs in deciding on resectability and treatment pathways. However, this risks the development of a postcode lottery in terms of which treatments can be offered to which patients, based on the expertise of local teams, capacity, and equipment/technology. One example of this is the long wait times for radiotherapy delivered at specialist centres. Immunotherapy is not a treatment that is emphasised in the guidelines; however, the recent developments in research into this area should be considered when updating guidelines in future. The sharing of knowledge, research, skills, and expertise will be key to improving care for patients with stage III N2 NSCLC in future.
